# Hybrids of Deep HOMO Organic Cyanoacrylic Acid Dyes and Graphene Nanomaterials for Water Splitting Photoanodes

**DOI:** 10.3390/ma18020463

**Published:** 2025-01-20

**Authors:** Alejandro Ansón-Casaos, Ana M. Benito, Wolfgang K. Maser, Jesús Orduna, Belén Villacampa, María-Jesús Blesa

**Affiliations:** 1 Instituto de Carboquimica, ICB-CSIC, 50018 Zaragoza, Spain; 2 Departamento de Química Orgánica, Universidad de Zaragoza, 50009 Zaragoza, Spain; 3 Instituto de Nanociencia y Materiales de Aragón (INMA), CSIC-Universidad de Zaragoza, 50009 Zaragoza, Spain; 4 Departamento de Física de la Materia Condensada, Universidad of Zaragoza, 50009 Zaragoza, Spain

**Keywords:** metal-free dye, donor-π-acceptor, photocatalyst, electrolysis, hydrogen

## Abstract

Dye-sensitization is a promising strategy to improve the light absorption and photoactivity abilities of wide-bandgap semiconductors, like TiO_2_. For effective water-splitting photoanodes with no sacrificial agents, the electrochemical potential of the dye must exceed the thermodynamic threshold needed for the oxygen evolution reaction. This study investigates two promising organic cyanoacrylic dyes, designed to meet that criterion by means of theoretical calculations. Both yellow-colored dyes were synthesized and characterized by optical and photoelectrochemical techniques, demonstrating strong light absorption in the visible region, suitable experimental reduction potentials, and adsorption from the organic solvent onto mesoporous TiO_2_ layers. In addition, to promote immobilization in aqueous electrolytes, the dyes were hybridized with graphene oxide or multi-walled carbon nanotubes. Photoelectrochemical analysis of the dye-sensitized photoelectrodes demonstrated efficient charge transfer from the dyes to the TiO_2_ photoanode under simulated solar light. While the starting photocurrent notably surpassed the blank TiO_2_, a subsequent decay points to kinetic obstacles that still need to be overcome.

## 1. Introduction

The photoactivity of wide band-gap metal-oxide semiconductors under solar irradiation is restricted by their low absorption in the visible range [1,2]. Sensitization with organic dyes has proved to be a suitable solution to improve the usable spectral window in electrochemical photovoltaics, giving rise to the so-called dye-sensitized solar cells (DSSCs), or sometimes Graetzel cells. Following a similar concept, numerous attempts have been made to construct other dye-sensitized photoelectrochemical (PEC) systems, in particular for water splitting towards the production of hydrogen. Many examples, derived from the former success in DSSCs, are based on organometallic centers, often ruthenium complexes [3,4,5,6].

Research on metal-free organic sensitizers has been gaining impulse with the aim of avoiding scarce elements. Families of dyes that have been tested in PEC water splitting include donor-π-spacer-acceptor (D-π-A) dyes [7,8,9]. In D-π-A molecules, the donor group works as the electron source after its photoexcitation, starting the flow of charges towards the acceptor part (A) and through the conjugate π-bridge. Electron-donor groups such as coumarin, phenothiazine, carbazole, indole, and triphenylamine (TPA), among other units, have been used [8]. Also, *N*, *N’*-dialkylanilines offer good sensitization capabilities due to their good light absorption, which is manifested by the intramolecular charge transfer (ICT) band that shifts towards the red compared to the commonly used TPA derivatives [10,11]. Suitable π-spacers are based on thiophene heteroaromatic ring systems since they offer dye stability and an extended π-conjugation, improving the molar extinction coefficient while preventing the degradation of the oxidized form of the dye in water [12]. Finally, a favorable acceptor system is commonly based on cyanoacetic groups, whose electron-withdrawing properties are provided by the cyano part, while bonding possibilities with TiO_2_ are provided through the carboxylic group [13]. However, the hydrolyzation of the ester bond between the anchoring group and TiO_2_ may limit the dye performance in aqueous environments or in alkaline water-splitting conditions.

All the organic dyes face the difficulty of stability in aqueous media, in particular under water oxidation conditions in a photoanode [14]. Actually, most studies on dye-sensitized photoanodes are performed with a sacrificial reagent in the electrolyte, such as triethanolamine, which is more easily oxidized than water and facilitates dye stability [7]. In theory, a first basic requirement to prevent oxidative degradation under pure water oxidation conditions is a dye potential higher than the potential for oxygen reduction (+1.23 V vs. NHE). Strategies to improve chemical stability include molecular design [15,16], protective groups and layers [17], as well as boosting the desired charge transfer kinetics by means of co-catalysts [18,19].

Graphene materials have been applied in the photoanode of DSSCs and PEC water-splitting cells, mixing optimal quantities with TiO_2_ and forming hole transport layer coatings [20,21,22,23,24]. As a general trend, improvements in photocurrents and solar conversion efficiencies have been obtained for relatively small loadings (around 1 wt%) of the carbon nanomaterial in TiO_2_ [25]. A few examples have also demonstrated the possibilities of hybridization of graphene with photoactive organic components [26,27,28]. The transport and structural properties of carbon nanomaterials facilitate their use as supports for multifunctional hybrid platforms [28]. Typically, charge transfer complexes are quite easily formed between dye molecules and carbon nanomaterials [29,30].

In the present study, two cyanoacrylic molecules are proposed as potential dyes for sensitizing TiO_2_ photoanodes in water-splitting conditions. Importantly, this family of cost-effective, metal-free compounds has been seldom tested in these PEC experiments despite their straightforward synthesis. Guided by theoretical calculations, two promising candidates, with higher potential than oxygen, one novel, were identified. Moreover, none of them has been previously evaluated in PEC water splitting. Both dyes were synthesized, characterized, and hybridized with a small amount of a carbon nanomaterial, either graphene oxide (GO) or multi-walled carbon nanotubes (MWCNTs), to promote stability and prevent desorption in the aqueous electrolyte.

## 2. Materials and Methods

### 2.1. Density Functional Theory (DFT) Calculations

DFT calculations were performed using Gaussian 16 [31] with the ultrafine integration grid. Solvent (CH_2_Cl_2_) effects were estimated using a Conductor-like Polarizable Continuum Model (CPCM) [32,33]. Equilibrium geometries were optimized using the M06-2x hybrid meta-GGA exchange correlation functional [34] and the medium-size 6-31G* base [35]. Ground state, first excited state, and oxidized radical cation geometries were characterized as minima by frequency calculations.

Excitation energies were calculated by time-dependent single-point calculations using the M06-2x/6-311+G (2d,p) model chemistry. Absorption spectra were estimated through the calculation of vertical excitations at the ground state geometry using the equilibrium CPCM solvation. Ground state oxidation potentials (E_ox_), excitation energies (E_0-0_), and excited state oxidation potentials (E*_ox_) were determined from ΔG values, which in turn were obtained using the M06-2x/6-311+G (2d,p) energies and calculating the thermal corrections to Gibbs free energy at the M06-2x/6-31G* level.

Molecular orbital contour plots at the 0.04 isosurface value were obtained using the Avogadro 2 software [36].

### 2.2. Dye Synthesis and Characterization

The dye 2-cyano-3-(2,2′-bithiophen-5-yl)-acrylic acid (TT-CNCOOH) was synthesized following the protocol reported in the literature [37]. The structural characterization is included in Supplementary Materials (Figures S1–S8).

#### 2.2.1. (E)-3-(4-((2-(Tert-butyldimethylsilyloxy)ethyl)(methyl)amino)phenyl)-2-cyanoacrylic Acid (ASIL-CNCOOH)

A total of 0.09 g of cyanoacetic acid (1.02 mmol) was solved in 5 mL of dry CHCl_3_ at 0 °C, and subsequently 0.44 mL piperidine (4.5 mmol) was added drop by drop. Cyanoacetic acid (99%) was used as received, and piperidine (99%) was purified by redistillation; both were purchased from Sigma-Aldrich, Merck (Darmstadt, Germany). Next, 0.2 g of 4-({2-[(tert-butyldimethylsilyl)oxy]ethyl}(methyl)amino)benzaldehyde (ALD, synthesis details in the Supplementary Materials, Scheme S1) (0.6 mmol) was added, and the reaction was refluxed for 24 h under argon atmosphere in the dark. Afterwards, it was cooled down to room temperature. It was acidified with HCl 0.1 M for 30 min, brined, and washed with NH_4_Cl (1 × 60 mL) and H_2_O (2 × 60 mL). A dark yellow solid was obtained (40 mg, 16%).

Molecular weight (g/mol): 360. Melting point (°C) at 760 mm Hg: 116. IR (KBr) cm^−1^: 3454 (O-H), 2217 (C≡N). ^1^H-RMN (400 MHz, CDCl_3_) δ (ppm): 8.1 (s, 1H), 7.85 (s, 2H), 6.6 (s, 2H), 3.78 (s, 2H), 3.52 (s, H), 3.05 (s, 2H), 0.85 (s, 9H), 0.06 (s, 6H). ^13^C-RMN (100 MHz, CDCl_3_) δ (ppm): 170.0, 155.1, 152.7, 134.3, 119.9, 111.50, 60.6, 54.5, 39.6, 25.9, 18.3, −5.3.

#### 2.2.2. Molecular Optoelectronic Characterization

Optical and electrochemical techniques that were applied to dye molecules are included in the Supplementary Materials (Figures S9–S11).

### 2.3. Photoanode Preparation and PEC Tests

Fluorine-doped tin oxide (FTO) substrates (AGC, 80 Ω sq^−1^, 25 × 10 × 1.1 mm^3^) were purchased from Solems (Palaiseau, France). Substrates were thoroughly cleaned, and a TiO_2_ layer was deposited on a 1 cm^2^ area, applying a TiO_2_ paste (Sigma-Aldrich 791555) by screen printing. Finally, TiO_2_ layers were sintered at 500 °C for 15 min, following a slow heating ramp, specifically: up to 325 °C at 10 °C/min, which is maintained for 5 min; to 375 °C (5 °C/min) for 5 min; to 450 °C (5 °C/min) for 5 min; and to 500 °C (5 °C/min), maintaining it for 15 min.

The GO material was prepared using a modified Hummers method [30]. It was dispersed in water (0.1 mg·mL^−1^) and diluted in acetone to 0.5 × 10^−3^ mg·mL^−1^. The MWCNT powder (NC7000, Nanocyl, Sambreville, Belgium) was dispersed (1 mg·mL^−1^) in an aqueous solution of sodium dodecylbenzenesulfonate (SDBS, 2 mg·mL^−1^), and diluted in water to 10^−3^ mg·mL^−1^.

Dye molecules were dissolved in acetone at 0.1 mM. Next, a suitable volume of the carbon nanomaterial dispersion was added, achieving a 1% ratio of the dye mass. UV–vis spectra were measured in a Shimadzu UV-2401PC spectrophotometer (Kyoto, Japan). TiO_2_/FTO substrates were immersed in the liquid for 18 h, gaining a pale-yellow color.

PEC measurements were performed in a 3-electrode cell provided with a quartz window for irradiation. Working electrodes were prepared connecting the FTO surface to the wire with adhesive copper tape. The counter-electrode was a 6 mm graphite rod. The reference electrode was an Ag/AgCl (3M NaCl) from Metrohm (Herisau, Switzerland). The electrolyte was 0.1 M Na_2_SO_4_. All the measurements were performed in an analogous way: first, two complete conditioning voltammetry cycles at 20 mV·s^−1^ from 0.4 to −1.1 V were carried out in the dark; after that, transient photocurrent measurements were followed at a potential of *E* = 0 V vs. Ag/AgCl. Light irradiation (100 mW·cm^−2^) was provided by a solar simulator consisting of a 150 W xenon lamp, a solar filter, and the suitable optics by Quantum Design (Pfungstadt, Germany).

## 3. Results and Discussion

### 3.1. Theoretical Calculations

**Figure 1:**
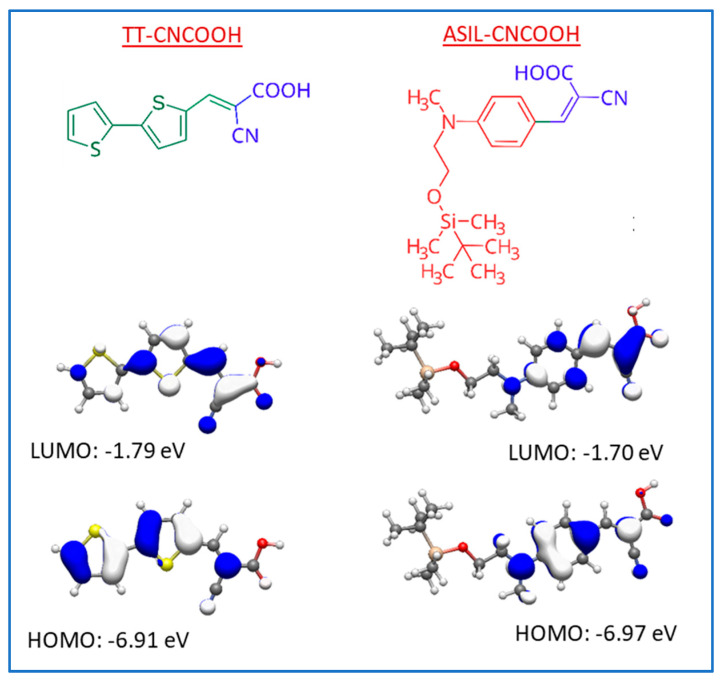
Chemical structure of the selected dye molecules TT-CNCOOH and ASIL-CNCOOH, and electronic density charts of their HOMO and LUMO levels with the corresponding energy values.

The structure of dye molecules that were selected for the present study, namely, ASIL-CNCOOH and TT-CNCOOH, is shown in Figure 1. Both molecules bear terminal cyanoacetic acid groups and have a simple molecular structure. Charts of electron density for the relevant HOMO and LUMO molecular orbitals are included in Figure 1, and the associated characteristic parameters are listed in Table 1. In particular, the calculated redox potentials (E_ox_) are +1.41 and +1.90 V vs. NHE, well above the potential of oxygen reduction (+1.23 V). Therefore, both organic molecules should be thermodynamically able to oxidize water to O_2_.

### 3.2. Synthesis and Optoelectronic Characterization of Dye Molecules

The synthesized low-molecular-weight dyes, being cyanoacrylic acid derivatives, were designed to study potential applications in photoelectrochemistry. These acid derivatives (ASIL-CNCOOH and TT-CNCOOH) were obtained using Knoevenagel condensation. Theoretically predicted properties (Table 1) can be compared with experimental measurements, specifically UV–vis spectroscopy (Table 2) and differential pulse voltammetry (DPV, Supplementary Materials, Figure S12).

**Figure 2:**
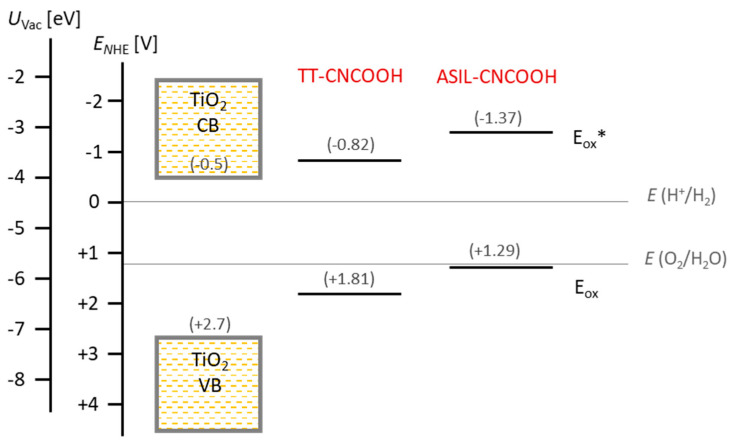
Diagram of experimental potential values of TT-CNCOOH and ASIL-CNCOOH. The estimated oxidation potential of the excited state of the dye was calculated as E_ox_* = E_ox_ − E_opt_.

Experimental oxidation potential values of the ground state (E_ox_) for dyes ASIL-CNCOOH and TT-CNCOOH (+1.29 V and +1.81 V) lie above those expected from DFT calculations (+1.41 and +1.90 V, respectively), but they are still below the oxygen reduction potential. This fact should thermodynamically enable the water-splitting process. The energies of the electronic levels are plotted in Figure 2. It can be observed that both dyes present nearly identical optical gaps (2.66 and 2.63 eV).

**Figure 3:**
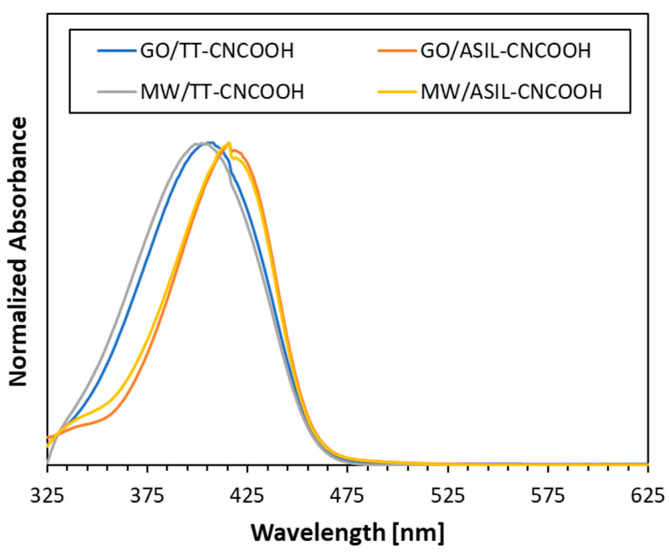
UV–vis spectra of carbon nanomaterial–dye mixtures in acetone.

The UV–vis absorption spectra of the dyes TT-CNCOOH and ASIL-CNCOOH with the carbon nanomaterials were carried out in acetone solvent (Figure 3). Both dyes present bands in the near UV–visible region between 350 and 450 nm. The spectra of the bithiophene dye (TT-CNCOOH) show a maximum λ_abs_ around 400 nm, whereas for the aniline derivative, λ_abs_ = 420 nm due to the donor effect of this ring. In both cases, absorption bands are attributed to the ICT between the bithiophene or the aniline part and the electron-withdrawing cyanoacetic group. No significant changes are observed upon the addition of a small quantity of different carbon nanomaterials, so only spectra of composite dyes are presented in Figure 3 for clarity. Notably, the carbon nanomaterials allowed an improved immobilization of dye molecules on the photoanode. Moreover, it will be shown that the presence of either GO or MWCNTs leads to distinct levels of photoactivity in the photoanode.

### 3.3. PEC Characterization of Dye-Sensitized TiO_2_ Photoanodes

**Figure 4:**
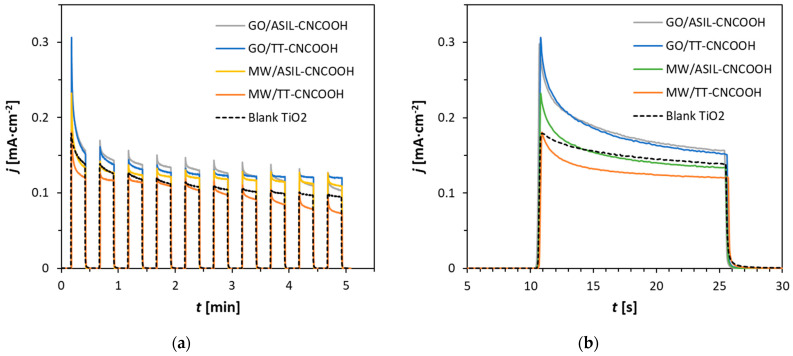
Transient photocurrent measurements in 0.1 M Na_2_SO_4_: (**a**) 10 on-off pulses; (**b**) magnified first pulse.

Sensitized photoelectrodes gained a yellowish color upon immersion in the carbon nanostructure–dye–acetone mixture. At the microscopic scale, no relevant changes in the electrode microstructure have been previously observed upon dye immersion [38]. It was observed that the presence of the carbon nanomaterial hindered dye detachment in the aqueous medium, allowing PEC measurements by chronoamperometry (Figure 4). Transient photocurrent experiments were performed in a nearly neutral pH, which has been widely probed in TiO_2_ and dye-sensitized TiO_2_ photoelectrodes [38,39]. The first irradiation period (Figure 4b) shows a different decay behavior of the reference TiO_2_ and dye-sensitized photoanodes. The starting photocurrent reaches high values on the sensitized electrodes, and it decays in a timeframe of 5 s to 10 s. This fact indicates that charge injection from the irradiated dye to the TiO_2_ electrode is hindered, probably due to a photo-oxidative degradation that result in a lowered photoactivity (Figure 4a).

The photocurrent of electrodes with GO hybrids was higher than that with MWCNTs, pointing to the effect of the small quantity of the carbon nanomaterial. Both TT-CNCOOH and ASIL-CNCOOH dyes showed similar behavior in the first irradiation pulse and after 5 min (Figure 4a), having lost their yellow color at the end of the experiment. While the observed degradation most likely is a result of the kinetic conditions of water oxidation, hindering rapid charge transfer for the regeneration of the organic dye molecule, it remains to highlight that both dye systems feature suitable thermodynamic potentials facilitating water splitting.

## 4. Conclusions

This study demonstrates the capability of two cyanoacrylic dyes, TT-CNCOOH and ASIL-CNCOOH, as promising sensitizers for TiO_2_ photoanodes in water oxidation. Their experimentally determined HOMO and LUMO levels agreed with those predicted by DFT calculations, confirming an adequate level alignment for the water oxidation process. The strategic hybridization of these dyes with carbon nanomaterials, such as GO and MWCNTs, significantly improved their immobilization, preventing the studied desorption of these kinds of dyes in the aqueous electrolyte. Furthermore, the obtained hybrids revealed successful charge injection from the dye to TiO_2_ under simulated solar irradiation during PEC experiments, with the GO-based hybrid being the most efficient. While the deep-lying HOMO levels of the dyes ensured thermodynamic feasibility for water oxidation, the rapid decay of the photocurrent within the initial 10 s of irradiation highlighted the need for further optimization. Future research should focus on improving the kinetic stability of these dye-sensitized photoelectrodes, considering the ability of the donor in the design of new sensitizers. Overall, this work provides valuable insights into the design based on DFT calculations and the application of cyanoacrylic dyes for the development of efficient and sustainable PEC devices.

## Figures and Tables

**Table 1:** Theoretical parameters ^1^ of compounds ASIL-CNCOOH and TT-CNCOOH.

Dye	λ_abs_^2^ [nm]	f_osc_^3^	E_ox_^4^ [V]	E_0-0_ [eV]	E_ox_* ^5^ [V]
ASIL-CNCOOH	387	1.26	1.41	3.02	−1.61
TT-CNCOOH	416	1.08	1.90	2.71	−0.81

^1^ Calculated using the M06-2x/6-311+G(2d,p) model chemistry and the CPCM solvation model. ^2^ Equilibrium CPCM values. ^3^ f_osc_: oscillator strength (related to the area below the absorption band). ^4^ Referenced to Normal Hydrogen Electrode (NHE). ^5^ The oxidation potential of the excited state of the dye was calculated from E_ox_* = E_ox_ − E_0-0_.

**Table 2:** Experimental optical properties of compounds ASIL-CNCOOH and TT-CNCOOH in dichloromethane.

Dye	λ_abs_ [nm]	Ɛ [×10^4^ M^−1^ cm^−1^]	λ_cut_ [nm]	E_opt_^1^ [eV]
ASIL-CNCOOH	429	2.92 ± 0.11	465	2.66
TT-CNCOOH	421	2.41 ± 0.29	472	2.63

^1^ The optical gap was estimated from the absorption spectra: E_opt_ = 1239.84/λ_cut_.

## Data Availability

The original contributions presented in this study are included in the article/Supplementary Material. Further inquiries can be directed to the corresponding authors.

## Supplementary Materials

The following supporting information can be downloaded at https://www.mdpi.com/article/10.3390/ma18020463/s1, S1: experimental methods of molecular characterization; S2: synthesis of ALD; S3: structural characterization; S4: optical and electrochemical characterization.
