# Supplementary material for: Hybrids of Deep HOMO Organic Cyanoacrylic Acid Dyes and Graphene Nanomaterials for Water Splitting Photoanodes
Source: Materials (Basel). 2025 Jan 20;18(2):463. doi: 10.3390/ma18020463 (PMC11766561; doi:10.3390/ma18020463)
Supplement: Supplementary file 1 [file materials-18-00463-s001.zip › materials-3376581-supplementary.pdf]

Supplementary Materials

# Hybrids of deep HOMO organic cyanoacrylic acid dyes and graphene nanomaterials for water splitting photoanodes

**Alejandro Ansón-Casaos [1,*], Ana M. Benito [1], Wolfgang K. Maser [1], Jesús Orduna [2,3], Belén Villacampa [2,4] and María-Jesús Blesa [2,3*]**

## TABLE OF CONTENTS

**S1. Experimental methods of molecular characterization**

**S2. Synthesis of compound ALD**

*Scheme S1. Synthesis of aldehyde ALD*

**S3. Structural characterization**

Figure S1. $^{1}$H-NMR spectrum of compound ALD (400 MHz, $CDCl_3$).

Figure S2. $^{13}$C-NMR (APT) spectrum of compound ALD (100 MHz, $CDCl_3$).

Figure S3. FTIR spectrum of compound ALD.

Figure S4. HRMS (ESI$^+$) of compound ALD.

Figure S5. $^{1}$H-NMR spectrum of compound ASIL-CNCOOH (400 MHz, $CDCl_3$).

Figure S6. $^{13}$C-NMR spectrum of compound ASIL-CNCOOH (100 MHz, $CDCl_3$).

Figure S7. FTIR spectrum of compound ASIL-CNCOOH.

**S3. Optical characterization**

Figure S8. UV-vis absorption spectra of ASIL-CNCOOH and TT-CNCOOH in DCM

Figure S9. Concentration dependence of ASIL-CNCOOH dye. UV-vis Absorption spectra in DCM.

Figure S10. Concentration dependence of TTCNCOOH dye. UV-vis Absorption spectra in DCM.

**S4. Electrochemical characterization**

Figure S11. Differential pulse Voltamogramms: (a) TT-CNCOOH; (b) ASIL-CNCOOH.

## 1. Experimental methods of molecular characterization

*Melting points* were obtained on a Gallenkamp apparatus in open capillaries and are uncorrected.

*$^1$H and $^{13}$C-NMR spectra* were recorded on a Bruker AV400 at 400 MHz and 100 MHz respectively; δ values are given in ppm (relative to TMS) and *J* values in Hz. The apparent resonance multiplicity is described as s (singlet), br s (broad singlet), d (doublet), t (triplet), q (quartet) and m (multiplet). $^1$H-$^1$H COSY and $^1$H-$^{13}$C-HSQC experiments were recorded in order to establish peaks assignment.

*Electrospray mass spectra* were recorded on a Bruker MicroToF-Q spectrometer and on a Bruker TIMS-TOF; accurate mass measurements were achieved using sodium formate as external reference.

*UV-Visible spectroscopy* of dye molecules in dichloromethane (DCM) was performed with an UV-vis Cary 6000.

*Differential Pulse Voltammetry (DPV)* measurements were performed with a μ-Autolab ECO-Chemie potenciostat, using a glassy carbon working electrode, Pt counter electrode, and Ag/AgCl reference electrode. The experiments were carried out under argon, in DCM with $Bu_4NPF_6$ as the supporting electrolyte (0.1mol $L^{-1}$). Scan rate was 100 mV $s^{-1}$.

## 2. Synthesis of compound ALD

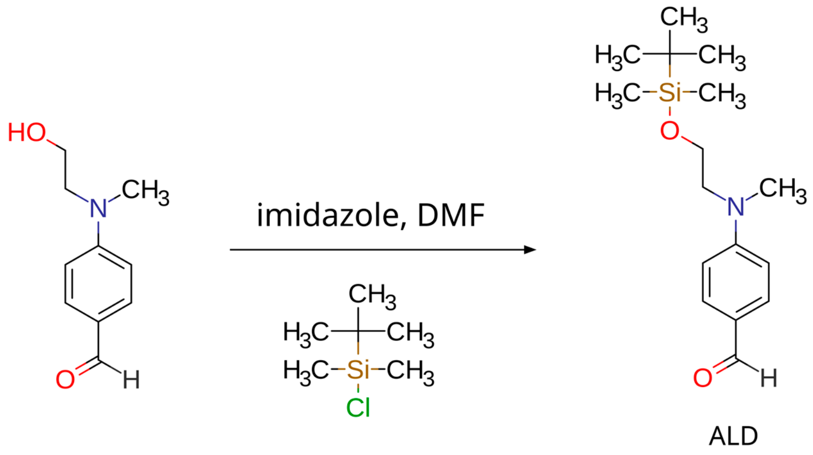


*Scheme S1. Synthesis of aldehyde ALD*

*4-({2-[(tert-butyldimethylsilyl)oxy]ethyl}(methyl)amino)benzaldehyde, ALD*

At 0°C, 0.25 g (3.75 mmol) of imidazole and 0.56 g (3.75 mmol) of *tert*-butyldimethylsilyl chloride was dissolved in 5 ml of dry *N,N*-dimethylformamide (DMF) under argon atmosphere. Next, 0.44 g (2.50 mmol) of 4-((2-hydroxyethyl)(methyl)amino)benzaldehyde was dissolved in DMF and introduced into the former solution. The reaction was maintained for 24 h at room temperature. The DMF was evaporated under reduced pressure, an oil being obtained. It was rinsed with ethyl acetate (3x60 mL). The organic phase was washed with NaCl (3x60mL) and a saturated $NH_4Cl$ solution (3x60mL). Finally, the crude was dried with $MgSO_4$, filtered and the solvent was removed. It was purified using column chromatography with hexane/AcOEt (80/20) eluent. Finally, 0.62 g of an orange oil (ALD) was obtained (Yield: 85%).

**Molecular weight**: 293.05 g/mol. **FTIR** (KBr) $cm^{-1}$: 1683 (C=O), 1114 (Si-H). **$^1$H-RMN** (400 MHz, $CDCl_3$) δ (ppm): 9.73 (s, 1H), 7.72 (d, *J*= 8.8 Hz, 2H), 6.72 (d, *J*= 8.8 Hz, 2H), 3.80 (t, *J*= 5.8 Hz, 2H), 3.60 (t, *J*= 5.8 Hz, 2H), 3.10 (s, 3H), 0.86 (s, 9H), 0.00 (s, 6H). **$^{13}$C-RMN** (100 MHz, $CDCl_3$) δ (ppm): 190.2, 153.6, 132, 125.1, 111, 60.4, 54.5, 39.5, 25.8, 18.2, -5.5. **HRMS** ($ESI^+$) m/z: calculated $[C_{16}H_{28}NO_2Si]^+$: 294.1889 found 294.1883$[M+H]^+$

## 3. Structural characterization

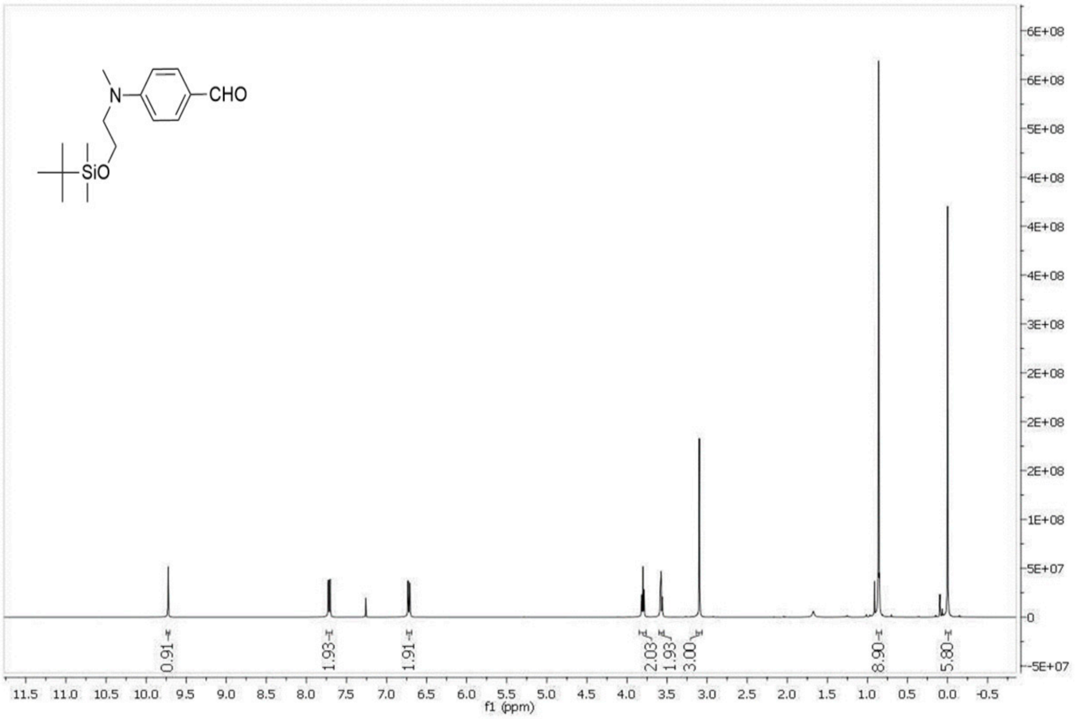


**Figure S1**. $^{1}$H-NMR spectrum of compound ALD (400 MHz, $CDCl_3$).

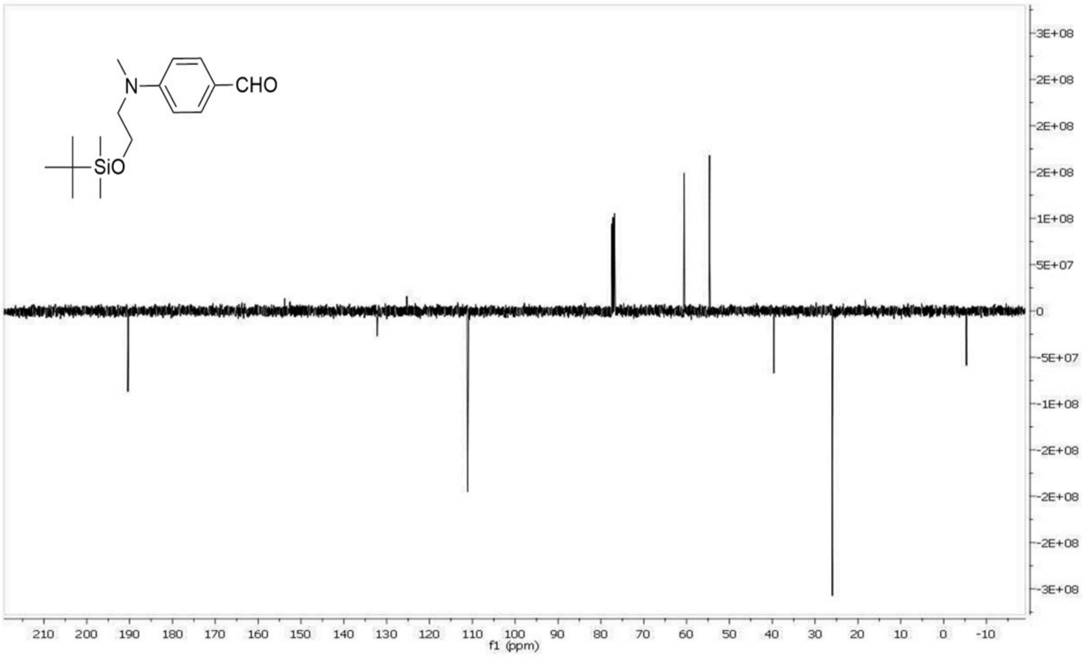


**Figure S2**. $^{13}$C-NMR (APT) spectrum of compound ALD (100 MHz, $CDCl_3$).

F

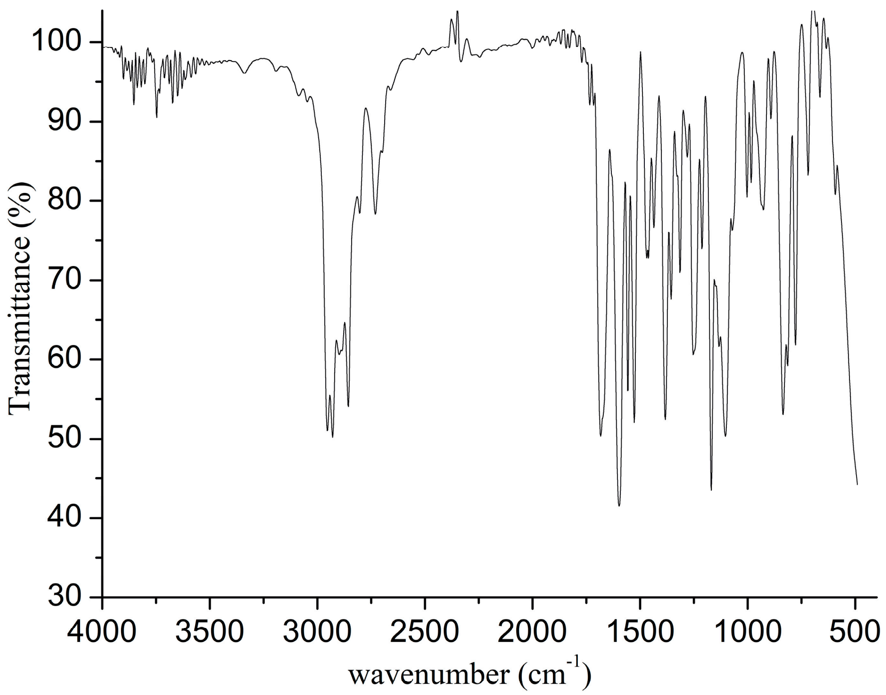


**Figure S3**. FTIR spectrum of compound ALD

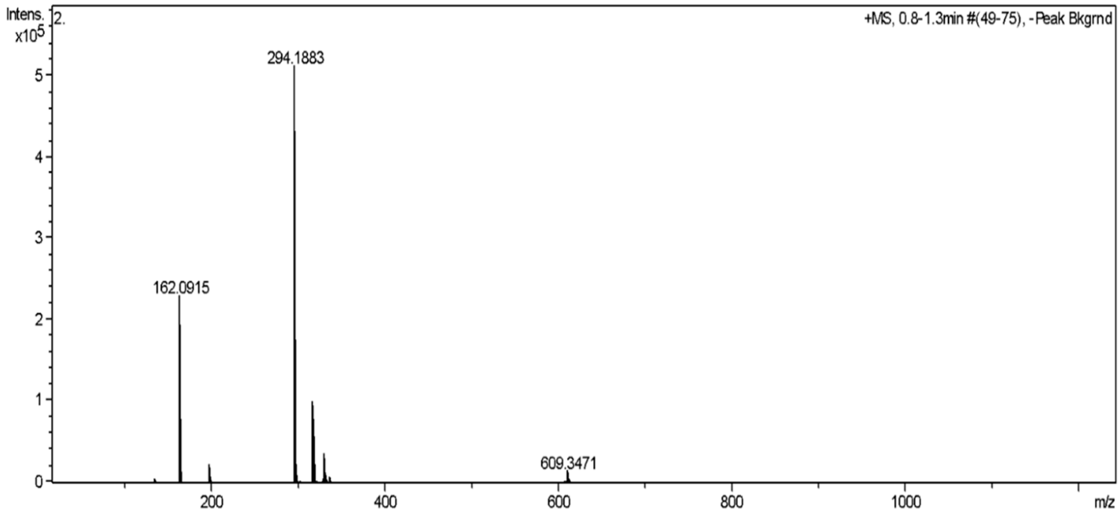


**Figure S4**. HRMS (ESI+) of compound ALD

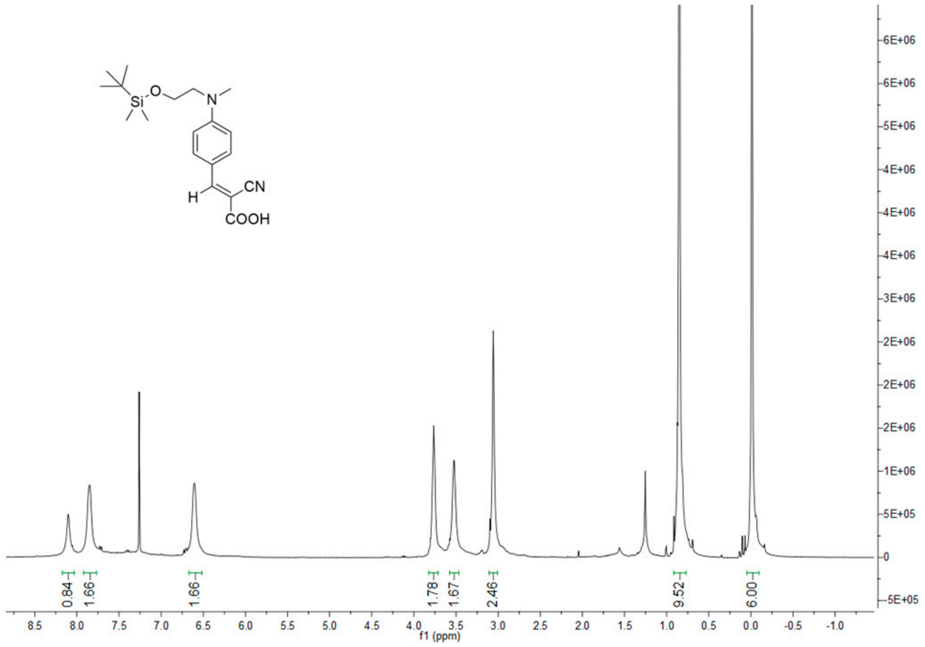


**Figure S5**. $^{1}$H-NMR spectrum of compound ASIL-CNCOOH (400 MHz, $CDCl_3$).

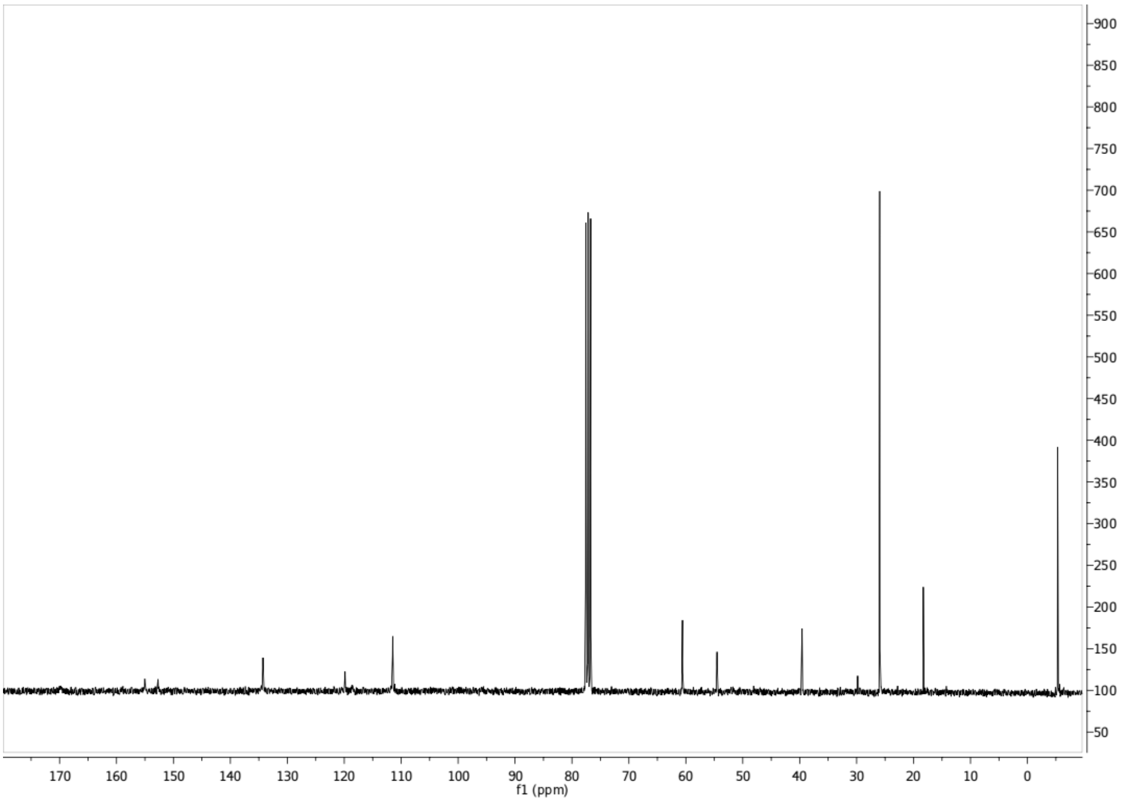


**Figure S6**. $^{13}$C-NMR spectrum of compound ASIL-CNCOOH (100 MHz, $CDCl_3$).

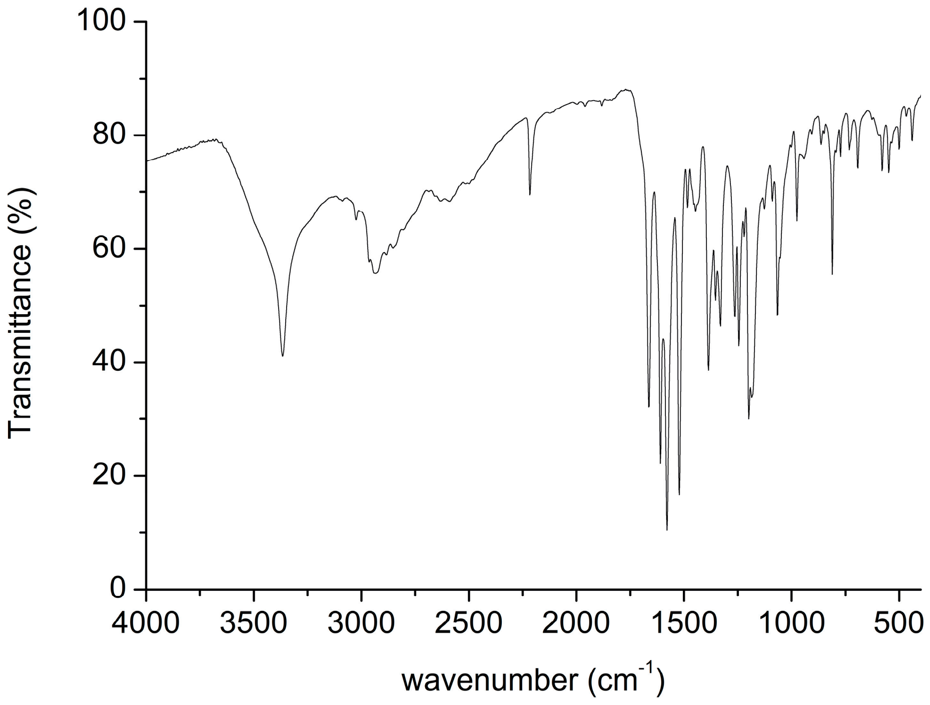


**Figure S7**. FTIR spectrum of compound ASIL-CNCOOH

## 4. Optical characterization

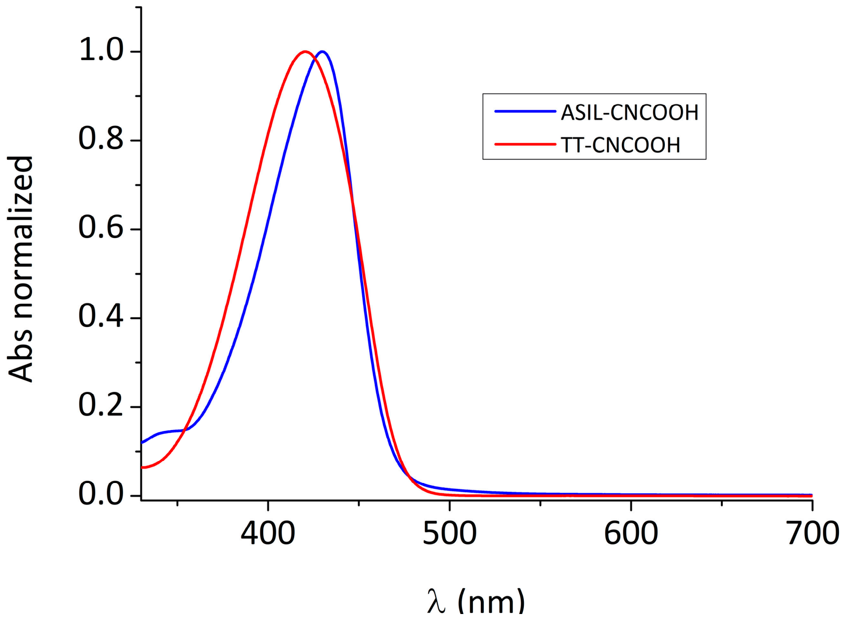


**Figure S8**. UV-Vis absorption spectra of ASIL-CNCOOH and TT-CNCOOH in DCM

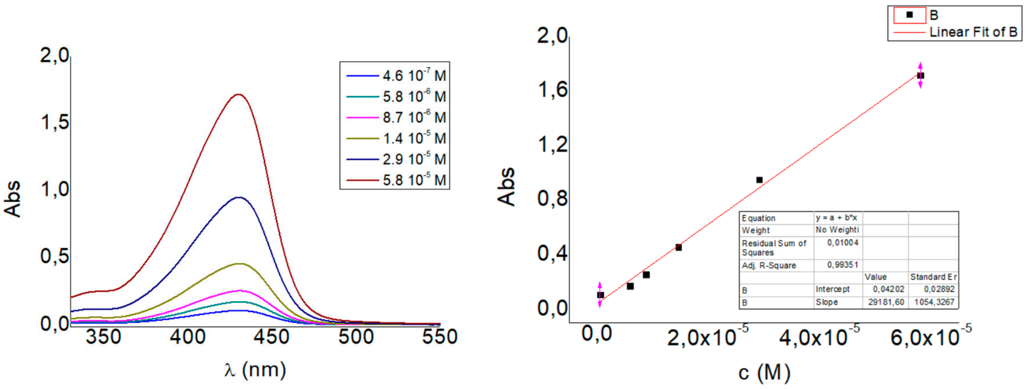


**Figure S9.** Absorption spectra in DCM. Concentration dependence of ASIL-CNCOOH dye. UV-vis

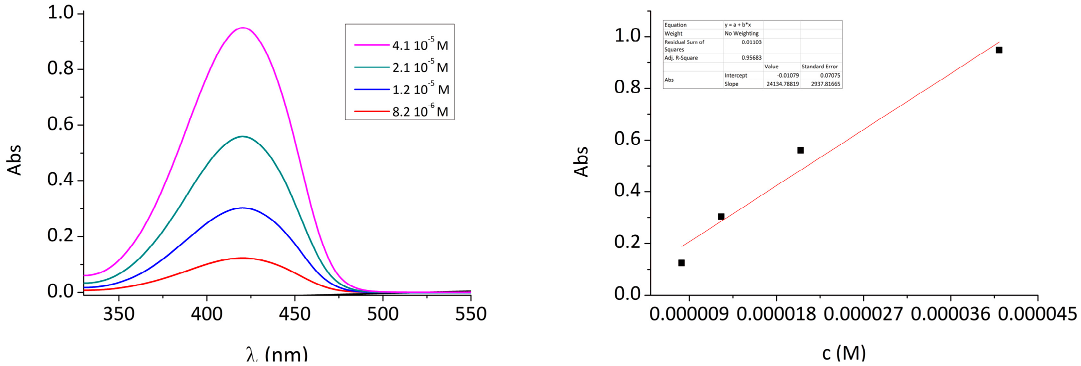


**Figure S10**. UV-vis Absorption spectra in DCM. Concentration dependence of TTCNCOOH dye.

## 4. Electrochemical characterization

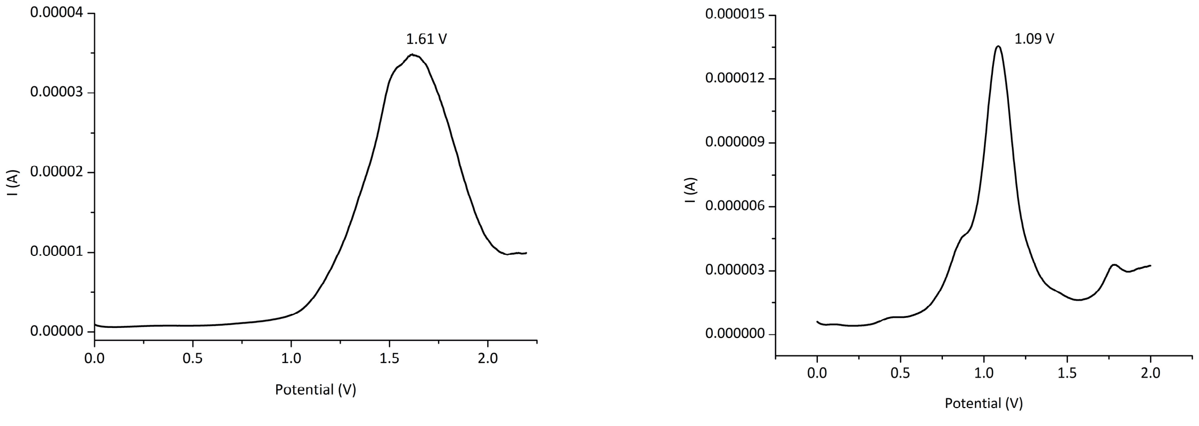


(a) (b)

**Figure S11**. Differential pulse Voltamogramms: (a) TT-CNCOOH; (b) ASIL-CNCOOH.
